# Efficacy of auriculotherapy in reducing pain: a comprehensive literature review

**DOI:** 10.3389/fpain.2026.1909832

**Published:** 2026-09-28

**Authors:** Miguel Faria, Paulo Sargento, Mónica Dias-Teixeira, Emidio Maia, Jorge Maia, José P. Pereira

**Affiliations:** 1 Instituto Politécnico da Lusofonia Escola Superior de Saúde Ribeiro Sanches, Lisbon, Portugal; 2 Faculty of Law, Lusofona University, Lisbon, Portugal; 3 Centro de Estudos Avançados em Direito Francisco Suarez, Universidade Lusófona, Lisbon, Portugal; 4 Rede de Quimica e Tecnologia Laboratorio Associado Para a Quimica Verde - Porto, Universidade do Porto, Porto, Portugal

**Keywords:** acupuncture, auricular, auriculotherapy, chronic, efficacy, pain, reduction, score

## Abstract

**Introduction:**

This review examines the efficacy of auriculotherapy in reducing pain across acute and chronic contexts, focusing on pain intensity scores, analgesic requirements, and safety profiles.

**Methods:**

A comprehensive, English-language literature search was conducted across PubMed, b-On, Web of Science, Scopus, and SciELO using Boolean combinations of terms related to auriculotherapy, chronic and acute pain, and randomized trials (searches first run March 2026, re-run June 2026). Eligibility was defined *a priori* using a PICOS framework. From 280 initially identified records, 13 studies were included after duplicate removal and eligibility screening, and findings were synthesized narratively by pain type given substantial heterogeneity across designs. Methodological quality was assessed using the Cochrane risk-of-bias tool for the seven included primary RCTs and AMSTAR-2 for the five included systematic reviews/meta-analyses.

**Results:**

Auriculotherapy significantly reduced acute postoperative pain, with meta-analyzed Visual Analog Scale (VAS) reductions of −0.44 (95% CI: −0.72 to −0.17) at 24 h and an analgesic-requirement standardized mean difference of −0.88 (95% CI: −1.29 to −0.46). For chronic back pain, standardized mean differences ranged from −0.65 to −0.85. In migraine, VAS decreased by −0.65 (95% CI: −0.86 to −0.43) with attack frequency reduced by an MD of −0.49 (95% CI: −0.59 to −0.40). Cancer pain studies reported 36% VAS reductions at 2 months. Adverse events were minimal, limited to mild local reactions in less than 5%–8% of cases, with no serious events reported.

**Discussion:**

Auriculotherapy demonstrates moderate, consistent efficacy across pain types, likely mediated through autonomic modulation and endorphin release via somatotopic ear projections. A dose–response pattern emerged, with multiple sessions (2–3 weekly over 4–8 weeks) yielding sustained effects. Stronger benefits were observed in orthopedic perioperative settings and with needle-retention protocols. It concludes that auriculotherapy is a safe, viable non-pharmacological adjunct for pain management, while calling for large, diverse, standardized RCTs with longer follow-up and objective biomarker measures to strengthen the evidence base. Because these findings derive from a narrative synthesis of a heterogeneous mix of primary trials, they should be interpreted as indicative rather than definitive, particularly given the diversity of study designs and predominantly Asian/perioperative populations represented.

## 1 Introduction

Pain remains one of the most prevalent and debilitating health issues worldwide, affecting millions and contributing to substantial socioeconomic burdens through reduced productivity, increased healthcare costs, and diminished quality of life. Acute pain, such as experienced postoperatively, and chronic conditions like back pain or migraines disrupt daily functioning and often require multimodal management to address both symptoms and underlying contributors (1, 2). Conventional treatments, including opioids and non-steroidal anti-inflammatory drugs, provide relief but carry risks of dependency, gastrointestinal complications, and tolerance, prompting exploration of complementary therapies (3, 4).

Auriculotherapy, defined as the stimulation of specific acupoints on the external ear to modulate pain and related symptoms, draws from traditional Chinese medicine principles where the ear is viewed as a microsystem reflecting the body's organs and systems (5, 6). This approach, often involving needle insertion, acupressure, or semi-permanent devices at points like Shenmen or site-specific zones, has gained traction in Western clinical settings, particularly for its minimally invasive nature and potential to enhance endogenous pain-relief mechanisms such as endorphin release and autonomic modulation (7). Despite growing adoption in perioperative care, emergency departments, and chronic pain clinics, the evidence base remains fragmented, with systematic reviews highlighting promising but inconclusive results due to methodological variability and limited large-scale trials (1, 3, 8). This inconsistency underscores the need for a synthesized evaluation that integrates findings across pain types and contexts to clarify auriculotherapy's role (9). The present review examines the efficacy of auriculotherapy in reducing pain, focusing on quantitative outcomes like intensity scores and analgesic needs, while considering safety profiles and influencing factors such as intervention protocols and patient populations (8).

## 2 Methods

### 2.1 Search strategy

We performed a comprehensive search in five databases: PubMed, b-On, Web of Science, Scopus, and SciELO. In each database, searches combined the following terms using Boolean operators: (“auriculotherapy” OR “auricular acupuncture” OR “auricular acupressure” OR “ear acupuncture”) AND (“chronic pain” OR “acute pain” OR “postoperative pain” OR “migraine” OR “cancer pain”) AND (“randomized” OR “randomised” OR “randomized controlled trial” OR “systematic review” OR “meta-analysis” OR “trial”). In PubMed, the free-text string was combined via AND with the MeSH term “Acupuncture, Ear” [Mesh]. Searches were restricted to English-language records, with no lower publication-date limit given the relatively small evidence base for this intervention. Searches were first run in March 2026 and re-run in June 2026 prior to submission to capture newly indexed records.

### 2.2 Study selection

Initial database searching identified 280 records. After duplicate removal and relevance-based filtering, 103 records were screened against eligibility criteria. Of these, 84 papers without full-text were excluded, resulting in 19 full text papers, of which 6 were excluded (see Table 1), therefore 13 studies were included in the final synthesis (see Figure 1).

**Table 1:** Full-text excluded studies.

Record (author, year)	Stage	Exclusion category	Reason for exclusion (mapped to PICOS, Section 2.2)	Source
Hou X, Xiong W, Lin X, Zhu Y, Yang R, Huang J, Chen Z, Ma H. (2021). Auricular acupuncture for shoulder pain: a protocol for systematic review and meta-analysis.	Full text	Wrong study design/publication type	Study protocol only (PROSPERO CRD42021238797); no completed results or outcome data have been published—fails the RCT/CCT/SR/MA “evaluating efficacy” criterion, which requires reported findings.	Medicine (Baltimore); PMID 33907132
Jackson HJ, Peal K. (2022). Auricular acupuncture for the treatment of cancer-related pain. Oncology Issues, 37 (4), 32–35.	Full text	Wrong study design/publication type	Non-systematic clinical practice/quality-improvement commentary describing a single institution's program; not a primary RCT/CCT or a systematic review/meta-analysis.	Oncology Issues; DOI 10.1080/10463356.2022.2079353
Wu TP, Chen FP, Liu JY, Lin MH, Hwang SJ. (2007). A randomized controlled clinical trial of auricular acupuncture in smoking cessation. Journal of the Chinese Medical Association.	Full text	Wrong outcome	Primary outcome is smoking cessation/nicotine withdrawal symptoms; no pain outcome reported despite meeting all other RCT/intervention criteria.	J Chin Med Assoc; PMID 17698433
Zhou J, Li S, Wang Y, Lei Y, Foreman RD, Yin J, Chen JD. (2017). Effects and mechanisms of auricular electroacupuncture on gastric hypersensitivity in a rodent model of functional dyspepsia.	Title/abstract	Wrong population	Animal (rat) model, not human participants; mechanistic study rather than a clinical trial.	PLoS One; PMID 28350818
Fu Q, Xie H, Zhou L, et al. (2021). TCM auricular point acupressure for the relief of pain, fatigue, and gastrointestinal adverse reactions after novel coronavirus-19 vaccine injection: structured summary of a study protocol.	Full text	Wrong study design; wrong population	Study protocol only, no completed results; primary condition is post-vaccination adverse reactions rather than the acute/chronic clinical pain populations targeted by this review's PICOS.	Trials; DOI: 10.1186/s13063-021-05138-3
Wu S, Liang J, Zhu X, Liu X, Miao D. (2011). Comparing the treatment effectiveness of body acupuncture and auricular acupuncture in preoperative anxiety treatment	Full text	Wrong outcome; partially wrong intervention	Primary outcome is preoperative anxiety, not pain; one trial arm uses body acupuncture rather than auricular acupuncture/acupressure/needling.	J. Med. Sci. PMID 21448381

**Figure 1:**
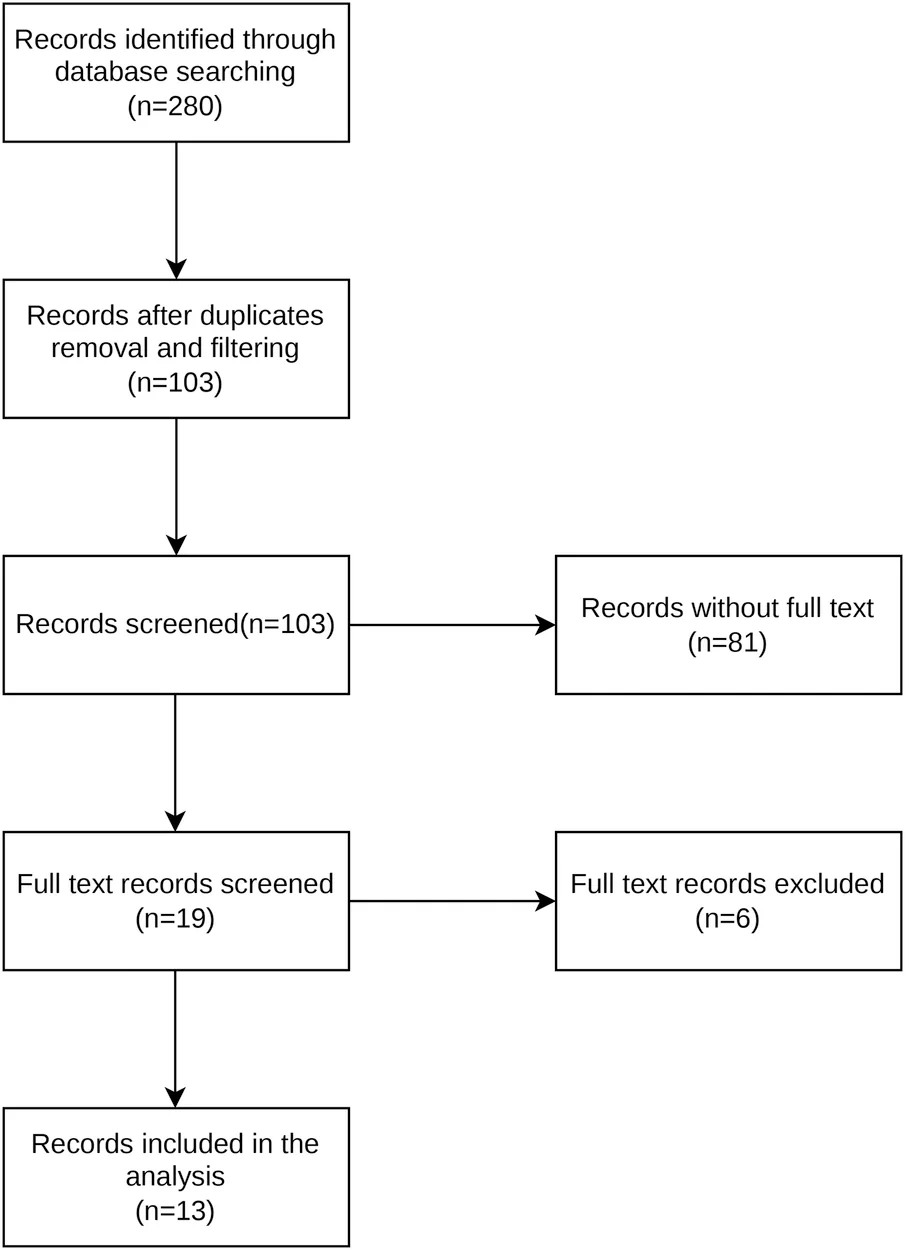
PRISMA flow diagram of studies selection.

Eligibility was defined *a priori* using a PICOS framework:

- Population: adults (≥18 years) with acute or chronic pain of any etiology.
- Intervention: auriculotherapy in its most common forms (needle, semi-permanent needle/seed, acupressure, or laser stimulation of auricular acupoints).
- Comparator: sham/placebo auriculotherapy, usual care, or active pharmacological/non-pharmacological comparators.
- Outcomes: pain intensity or reduction (e.g., VAS, NRS) as a primary or secondary outcome; analgesic consumption and adverse events considered as secondary outcomes where reported.
- Study design: randomized controlled trials, controlled clinical trials, or systematic reviews/meta-analyses evaluating efficacy.

### 2.3 Data extraction and synthesis

Data extraction focused on the following variables: study design (e.g., RCT), sample size, type of pain (acute/chronic, location), intervention method; auriculotherapy protocol: points stimulated, method (needle, acupressure), number of sessions, duration; control or comparison group (e.g., sham auriculotherapy, usual care, placebo); pain outcomes: primary pain measures (e.g., VAS, NRS scores), mean changes, effect sizes, statistical significance, and adverse events. Mixed study types were intentionally included because they serve complementary evidentiary roles: primary RCTs and RCT pilots provide direct, study-level effect estimates; the included systematic reviews and meta-analyses provide higher-level, pooled effect estimates drawn from larger aggregated samples addressing the same clinical questions; and the scoping review (7) contributes mechanistic and safety context not captured by effect-size-focused trials. Because these evidence types differ substantially in design, risk-of-bias profile, and level of aggregation, they were not statistically pooled into a single quantitative effect estimate; instead, findings were synthesized narratively and organized thematically by pain type (Section 2.5). We note that some primary RCTs included directly in this review [e.g., (10, 11)] also contribute data to one or more of the included meta-analyses [e.g., (9)], creating potential overlap in the underlying evidence base; this overlap, and its implications, is addressed further as a limitation in Section 4.3.

### 2.4 Risk of bias

Methodological quality was assessed using tools appropriate to each study design. For the seven primary randomized controlled trials and RCT pilots (10, 12–17), risk of bias was assessed by two authors, MT and MF, regarding random sequence generation, allocation concealment, blinding of participants and personnel, blinding of outcome assessment, incomplete outcome data, selective reporting, and other types of bias, as stated in the Cochrane Handbook for Systematic Reviews of Interventions (18), with each domain rated low, unclear, or high. For the five included systematic reviews and meta-analyses (9, 11, 14, 19, 20), methodological quality was instead assessed using AMSTAR-2 (A MeaSurement Tool to Assess systematic Reviews 2), which rates overall confidence in the results of each review as high, moderate, low, or critically low across 16 domains, including protocol registration, adequacy of the literature search, and assessment of publication bias. The scoping review (7) was appraised narratively for methodological transparency rather than rated with either instrument, consistent with its exploratory, mechanism-focused purpose. In case of disagreement between the two raters, a third author reviewer took the final decision.

### 2.5 Thematic findings

Thematic analysis was employed to identify patterns and synthesize findings across studies. Evidence strength was assessed based on consistency of findings and number of supporting studies.

Formal subgroup analyses (e.g., meta-regression stratified by intervention modality, stimulation duration, participant ethnicity, or pain chronicity) were not performed in this review. This decision reflects the substantial heterogeneity across the included evidence base, which spans primary RCTs, RCT pilots, systematic reviews, meta-analyses, and a scoping review with differing units of analysis, risk-of-bias profiles, and levels of aggregation (Section 2.3); combining these into quantitative subgroup strata would risk conflating study-level and pooled-review-level effect estimates and overstating the precision of any resulting comparison. Where the included evidence permits, narrative subgroup contrasts are instead offered within the thematic synthesis (Section 3.3) and discussion (Section 4.1)—for example, comparing retained-needle vs. single-session protocols, or seed/press-needle vs. indwelling-needle approaches—but these narrative comparisons should not be interpreted as formal, statistically adjudicated subgroup analyses.

## 3 Results

### 3.1 Characteristics of included studies

The included studies encompass a mix of randomized controlled trials, systematic reviews, meta-analyses and scoping reviews, spanning acute postoperative pain, chronic musculoskeletal and cancer pain. Sample sizes vary from small pilots (*n* = 20–87) to large aggregated meta-analyses (*n* = 766–1,527), with participants primarily adults in clinical settings such as perioperative, oncology, and primary care. Interventions consistently involve ear point stimulation via needles or acupressure, compared to sham, usual care, or placebo, with outcomes focused on pain scales like VAS/NRS (see Table 2).

**Table 2:** Characteristics of the included studies.

Author (Year)	Study Type	Sample Size	Pain Type	Intervention Method	Comparator	Primary Outcome Measure
Usichenko et al. (11)	Systematic review of RCTs	Aggregated from 9 RCTs (sizes not detailed)	Acute postoperative (abdominal, orthopedic)	Indwelling/semi-permanent needles at Shenmen, zero point	Sham acupuncture, usual care	VAS/NRS pain intensity; analgesic requirements
Zhou et al. (9)	Systematic review and meta-analysis of RCTs	1,527 (from 21 RCTs)*	Acute perioperative (abdominal, orthopedic, gynecological)	Needle insertion, acupressure, laser at Shenmen, sympathetic points	Sham, placebo, conventional care	VAS/NRS at 24 h; analgesic consumption
Elliott et al. (7)	Scoping review	Aggregated from 152 studies (>3 patients each)	Acute and chronic (postoperative, headache, musculoskeletal)	Indwelling needles, acupressure at Shenmen, zero point	Sham, usual care, placebo	VAS/NRS, McGill Pain Questionnaire
Goertz et al. (12)	RCT pilot	87	Acute syndromes (various)	Needle insertion (points not detailed)	Standard emergency care	NRS change from baseline
Moura et al. (21)	Systematic review and meta-analysis of RCTs	Aggregated from 15 RCTs (7 in meta-analysis)	Chronic back pain	Needle/acupressure at Shenmen, kidney, back points	Sham, usual care, placebo	VAS/NRS pain intensity
Moura et al. (14)	RCT	110	Chronic back musculoskeletal	Needle insertion at back-related points	Sham, no intervention	Brief Pain Inventory; algometer threshold
Zheng et al. (19)	Systematic review and meta-analysis of RCTs	766 (from 10 RCTs)*	Chronic migraine (episodic/chronic)	Needle/acupressure at Shenmen, occipital points	Sham, pharmacological, usual care	VAS; migraine days
Usichenko et al. (10)	RCT	120	Acute postoperative knee	Indwelling needles pre-surgery	Invasive sham	Ibuprofen requirement; VAS <40 mm
Alimi et al. (17)	RCT	90	Chronic cancer	Needle at electrodermal points (2 courses)	Sham acupuncture, sham seeds	VAS decrease at 2 months
Monteiro et al. (20)	Systematic review	Aggregated from 11 studies	Chronic cancer	Needles/seeds at Shenmen, sympathetic points	Sham, usual care, placebo	VAS/NRS pain intensity
Usichenko et al. (13)	RCT pilot	20	Acute postoperative knee	Press needles at Lung, Shenmen, Knee points	Sham at non-points	Analgesic quantity; VAS
Garner et al. (15)	RCT	45	Chronic (unspecified) with insomnia	Semi-permanent needles (standard protocol)	Usual care	Brief Pain Inventory severity/interference
Wang and Kain (16)	RCT	55	Anxiety (proxy for pain modulation)	Press needles at Shenmen/relaxation points (48 h)	Sham point	State anxiety score; physiological measures

### 3.2 Risk of bias

Cochrane risk-of-bias results reported below apply only to the seven primary RCTs/RCT pilots, consistent with the revised methodology in Section 2.4. All seven mentioned randomization. Three studies presented high risk in allocation, one incomplete outcome data, and another presented high risk in selective reporting (see Figure 2). Figure 3 shows the risk-of-bias assessments as a whole for the primary RCTs included in this review. AMSTAR-2 overall confidence ratings for the five included systematic reviews/meta-analyses are summarized in Table 3; the scoping review (7) is appraised narratively only, for the reasons given in Section 2.4.

**Figure 2:**
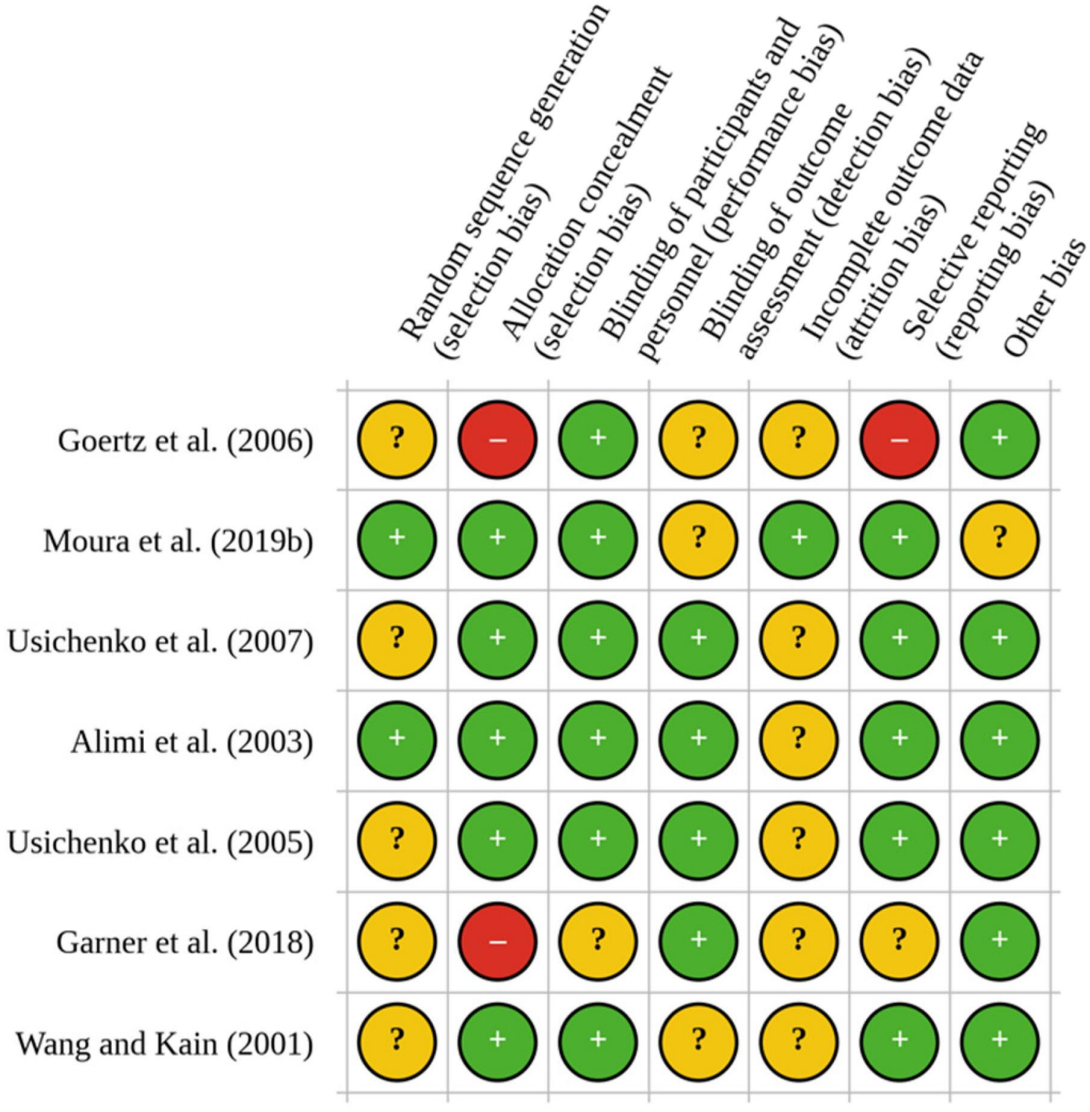
Risk of bias summary: review authors' judgements about each risk of bias item (primary RCTs only).

**Figure 3:**
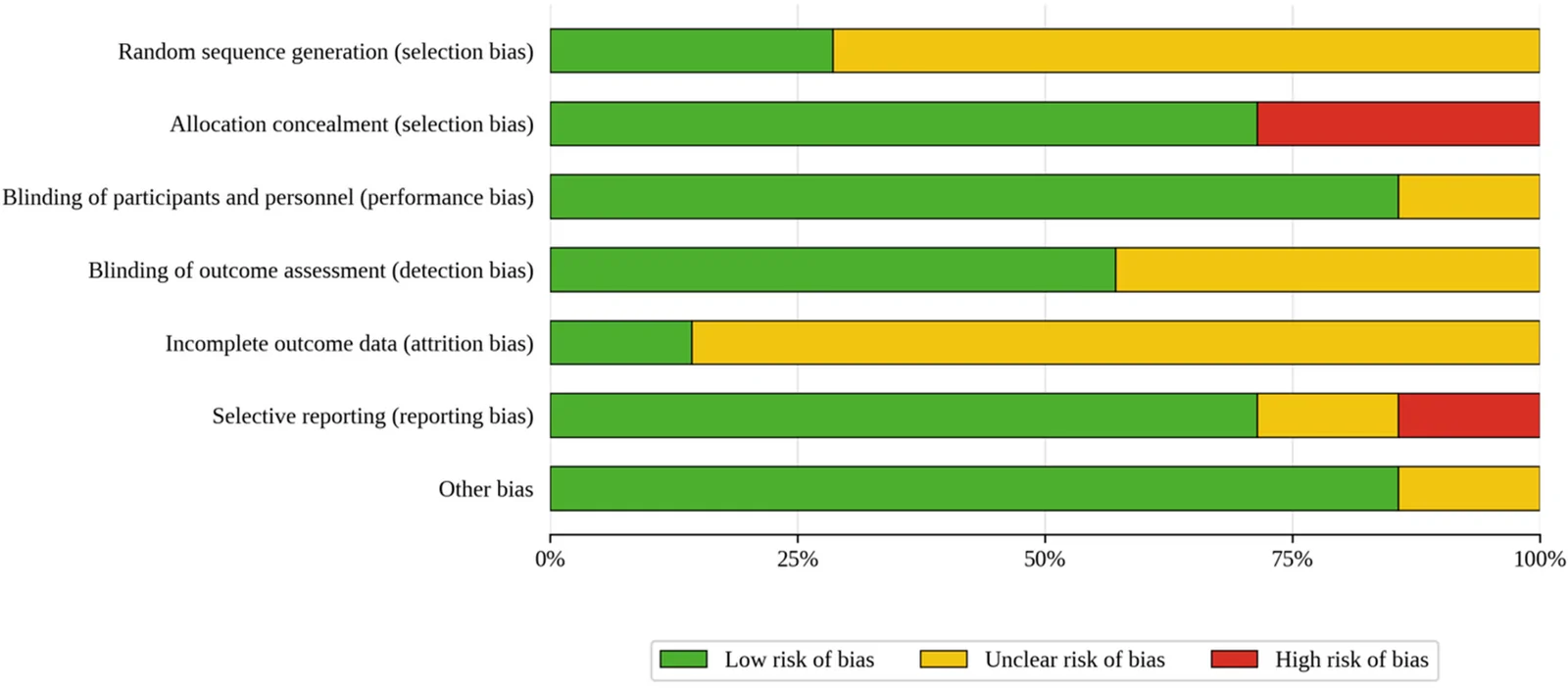
Risk of bias graph: review authors' judgements about each risk of bias item presented as percentages across the primary RCTs included in this review.

**Table 3:** AMSTAR-2 overall confidence ratings for included systematic reviews/meta-analyses.

Included Systematic Review/Meta-Analysis	Primary Basis for Rating	AMSTAR-2 Overall Rating
Usichenko et al. (11)	Predates PROSPERO/PRISMA (search to Sept 2007); quality rated with the Jadad scale rather than a domain-based RoB tool; narrative synthesis only, no meta-analysis; no registered protocol; no excluded-studies list.	Critically Low
Zhou et al. (9)	Strong on most domains: PROSPERO-registered, dual screening/extraction, Cochrane RoB, appropriate random-effects meta-analysis, heterogeneity explored, funnel-plot publication-bias assessment. Critical gap: no itemized list of full-text exclusions with reasons.	Low
Moura et al. (21)	No protocol registration identified; publication bias not assessed (defensible given only 7 meta-analyzed studies, but not discussed); no excluded-studies list; dual-review process not confirmed from available text.	Critically Low
Zheng et al. (19)	PROSPERO-registered, dual screening/extraction, RoB2, GRADE, heterogeneity explored—strong overall. But two critical domains unmet: no itemized excluded-studies list, and publication bias explicitly not assessed (authors note <10 studies per outcome, methodologically defensible but still an unmet AMSTAR-2 criterion).	Critically Low
Monteiro et al. (20)	No protocol registration identified; narrative synthesis only; risk-of-bias tool and dual-review process not confirmed from the sections available online.	Critically Low

### 3.3 Thematic findings

#### 3.3.1 Efficacy in acute postoperative and perioperative pain

Auriculotherapy significantly reduces acute postoperative pain intensity and analgesic requirements, with meta-analyzed mean differences in VAS scores of −0.44 (95% CI: −0.72 to −0.17, *p* < 0.001) at 24 h and a standardized mean difference of −0.88 (95% CI: −1.29 to −0.46) for analgesic requirement [(3), 21 RCTs, *n* = 1,527]. In emergency settings, a 23% NRS reduction from baseline (*p* < 0.0005) was observed immediately post-treatment, though effects equalized at 24 h (12). For ambulatory knee surgery, indwelling needles lowered ibuprofen use to a median of 200 mg (IQR 0–600) vs. 600 mg (IQR 200–800) in sham controls (*p* = 0.012), with VAS maintained below 40 mm similarly across groups (10); a pilot confirmed lower median consumption of 500 mg vs. 800 mg (*p* = 0.043) (11). Subgroup analyses showed a significant analgesic effect specifically for preoperative intervention and for orthopedic surgery, though the source meta-analysis did not report a separate pooled effect size for this subgroup (9). Outcomes were measured via VAS/NRS and analgesic proxies. Heterogeneity arises from protocol variations (e.g., single vs. retained needles), but positive directions prevail. Confidence: Moderate.

#### 3.3.2 Efficacy in chronic musculoskeletal and back pain

For chronic back pain, auriculotherapy yields significant VAS reductions with mean differences of −1.2 (95% CI: −2.1 to −0.3, *p* < 0.05) and SMD −0.65 across meta-analyzed trials, effective in 80% of studies for intensity and quality of life [Moura et al. (14)]; another synthesis reported SMD −0.85 (95% CI: −1.23 to −0.47, *p* < 0.001) (14). In a three-arm RCT, Brief Pain Inventory scores decreased significantly from baseline to final evaluation (*p* < 0.05) and 15-day follow-up (*p* < 0.05) in treatment and sham groups, with lower interference in treatment (*p* < 0.05), though no threshold change via algometer (21). For comorbid chronic pain and insomnia, Brief Pain Inventory severity and interference scores reduced significantly within and between groups over 8 days (15). Protocols involved 1–12 sessions of needle/acupressure at Shenmen/kidney points over 4–8 weeks, compared to sham/usual care. Effects are moderate to large, with *p* < 0.05 in 80% of cases, but lack of standardization contributes to variability; longer durations enhanced benefits (14). No population mismatches noted. Confidence: Moderate (generally consistent but limited by heterogeneous protocols).

#### 3.3.3 Efficacy in chronic migraine and headache pain

Meta-analyses indicate auriculotherapy reduces migraine VAS by −0.65 (95% CI: −0.86 to −0.43, *p* < 0.001), attack frequency by an MD of −0.49 (95% CI: −0.59 to −0.40, *p* < 0.001), and duration by MD −0.58 (95% CI: −0.64 to −0.52, *p* < 0.001), with relative risk for clinical effectiveness of 1.17 (95% CI: 1.10 to 1.25, *p* < 0.001) [(19), 10 RCTs, *n* = 766]. Protocols used weekly sessions of needles/seeds at Shenmen/occipital points, vs. sham/pharmacotherapy. Measurements via VAS show moderate-large effects (*p* < 0.001), assessed using RoB2 and GRADE (all outcomes rated moderate certainty). All included trials were conducted in China, limiting generalizability. Confidence: Moderate (consistent meta-analytic support with a registered protocol and RoB2 assessment, but geographically limited).

#### 3.3.4 Efficacy in cancer-related pain

Auriculotherapy decreases cancer pain VAS by 36% at 2 months (*p* < 0.0001) vs. 2% in placebo (17), with mean reductions of 1.5–3.5 points (0–10 scale, *p* < 0.05 in 8/11 studies) (20). Effects are moderate (Cohen's d 0.4–0.8), more pronounced with multiple sessions and in non-metastatic cases (20). Outcomes via VAS/NRS, with consistent positive directions but variable reporting. Confidence: Limited (sparse controlled trials, observational elements).

#### 3.3.5 Safety profile and adverse events

Adverse events are minimal across pain types, with mild local reactions like ear tenderness or erythema in 3%–5% (9), itching/bleeding in 8% for migraine (19), and soreness/dizziness <5% overall (7). No serious events or dropouts due to auriculotherapy; rates comparable to sham (20). In perioperative contexts, nausea/vomiting occurred rarely without attribution (9). No events reported in several trials (10–12). Safety supports adjunct use, with no population mismatches. Confidence: Strong (consistent low-incidence reporting).

#### 3.3.6 Summary of evidence

Table 4 presents the summary of evidences for the included studies. We can see that all reported positive aspects, with confidence levels that vary from limited to strong.

**Table 4:** Summary of evidences for the included studies.

Theme	Key Finding	Population Applicability	Effect Direction	Confidence Level	Supporting Studies
Acute Postoperative and Perioperative Pain	MD −0.44 (95% CI: −0.72 to −0.17, *p* < 0.001) in VAS; analgesic-requirement SMD −0.88 (95% CI: −1.29 to −0.46)	Adults undergoing surgery (abdominal, orthopedic)	Positive	Moderate (consistent findings with reasonable design quality)	Zhou et al. (9), Usichenko et al. (10), Goertz et al. (12), Usichenko et al. (11)
Chronic Musculoskeletal and Back Pain	SMD −0.65 to −0.85 (95% CI: −1.23 to −0.47, *p* < 0.001); significant BPI decrease (*p* < 0.05)	Adults with chronic back disorders	Positive	Moderate (generally consistent but limited by heterogeneous protocols)	Moura et al. (21), Moura et al. (14) Garner et al. (15)
Chronic Migraine and Headache Pain	MD −0.65 (95% CI: −0.86 to −0.43, *p* < 0.001) in VAS; RR 1.17 (95% CI: 1.10-1.25, *p* < 0.001)	Adults with episodic/chronic migraine (primarily Chinese)	Positive	Moderate (consistent meta-analytic support but geographically limited)	Zheng et al. (19)
Cancer-Related Pain	36% VAS reduction (*p* < 0.001); mean 1.5–3.5 point decrease (*p* < 0.05)	Adults with cancer pain	Positive	Limited (sparse controlled trials, observational elements)	Alimi et al. (17), Monteiro et al. (20)
Safety Profile and Adverse Events	Mild events in <5–8% (e.g., tenderness, no serious AEs)	Adults across pain types	Positive (low risk)	Strong (consistent low-incidence reporting)	Zhou et al. (9), Elliott et al. (7), Zheng et al. (19), Monteiro et al. (20)

## 4 Discussion

The synthesis reveals auriculotherapy's consistent capacity to attenuate pain across acute and chronic contexts, with meta-analytic evidence pointing to VAS reductions of −0.44 (95% CI: −0.72 to −0.17) in perioperative settings and −0.65 (95% CI: −0.86 to −0.43) for migraines, alongside SMDs of −0.65 to −0.85 for chronic back pain. These patterns emerge because auriculotherapy likely engages somatotopic ear projections that interface with central pain pathways, as suggested by scoping reviews linking it to autonomic nervous system modulation and neuroendocrine responses like endorphin release, which dampen nociceptive signaling more effectively in acute inflammatory states than entrenched chronic sensitization (7). When viewed collectively, the evidence uncovers a dose-response gradient where multiple sessions (e.g., 2–3 weekly over 4–8 weeks) yield sustained effects, such as 36% VAS drops in cancer pain persisting to 2 months, a trend invisible in isolated trials but indicative of cumulative neuromodulation (17).

Stronger perioperative benefits in orthopedic subgroups vs. general surgery may reflect targeted point stimulation aligning with localized inflammation, enhancing vagal tone to reduce analgesic requirements (SMD −0.88, 95% CI −1.29 to −0.46) (9). Confidence is highest for safety (minimal AEs <5%), grounded in uniform reporting across diverse designs, while efficacy claims are moderately confident due to consistent directions tempered by blinding challenges in acupuncture trials, which could inflate placebo-responsive outcomes like self-reported VAS. Mechanistic data, though limited to autonomic and neuroendocrine pathways, bolsters causality by connecting peripheral ear stimulation to central analgesia, yet gaps in biomarker assays (e.g., no cortisol or cytokine measures) leave physiological chains tentative. This review advances the field by highlighting protocol heterogeneity as a key variability source—needle retention up to 48 h outperforms single sessions—offering a synthesized framework for optimized application beyond individual study silos.

Beyond these physiological interpretations, it is worth noting that some behavioral outcomes reported in the included trials are conceptually adjacent to the psychological construct of pain acceptance—broadly, the capacity to engage in valued activity despite ongoing pain rather than prioritizing pain elimination or control. It is important to state clearly at the outset that no included study administered a validated pain-acceptance instrument (e.g., the Chronic Pain Acceptance Questionnaire, CPAQ), and none of the included trials set out to measure this construct. The following observations are therefore offered as a *post-hoc* interpretive hypothesis generated by the review authors, not as an empirical finding reported by any primary study, and should not be read as evidence that auriculotherapy affects pain acceptance.

With that boundary in place, two observations may be noted as hypothesis-generating rather than confirmatory. First, the reduction in postoperative analgesic consumption reported by Usichenko et al. (13) is not inconsistent with—though it does not demonstrate—a shift toward reduced reliance on pharmacological control. Second, the improvement in Brief Pain Inventory interference scores without a corresponding change in algometer-measured pain threshold (21) is compatible with, but does not establish, better functional tolerance of residual pain. Neither observation permits an inference about acceptance-based mechanisms, since both are equally explicable by ordinary analgesic and functional effects unrelated to the acceptance construct.

Formal verification of any relationship between auriculotherapy and pain acceptance would require future trials to prospectively administer validated instruments such as the CPAQ (or equivalent measures) as a defined outcome, rather than inferring the construct retrospectively from proxy measures such as analgesic consumption or interference scores. Until such data exist, this line of interpretation should be treated as speculative.

### 4.1 Comparison with existing literature and resolution of contradictions

Findings align with prior reviews emphasizing auriculotherapy's adjunctive value, such as reduced postoperative analgesics in military contexts, where 23% NRS drops mirror broader scoping evidence of 20%–50% opioid sparing through shared endorphin-mediated mechanisms that amplify standard care without additive risks (7, 12). This consistency underscores robustness, as converging meta-analyses (e.g., SMD −0.88 for perioperative needs) validate earlier pilots like the 2005 knee study (median 500 mg ibuprofen vs. 800 mg sham, *p* = 0.043), implying reliable signal despite evolving sham designs (9, 11).

Contradictions appear in ambulatory knee trials, where one RCT found no VAS superiority over invasive sham despite lower ibuprofen (200 mg median vs. 600 mg, *p* = 0.012) (10), contrasting positive meta-analytic trends; this likely stems from stringent pain thresholds (<40 mm VAS) masking subtle differences, as sham needling induced comparable placebo analgesia in short-term ambulatory recovery, unlike retained-needle protocols in meta-analyses that allowed prolonged stimulation. Another null for pain threshold via algometer in back pain RCT (no change, *p* > 0.05) despite BPI reductions (*p* < 0.05) (21) may reflect algometry's insensitivity to subjective interference vs. VAS's capture of multidimensional chronic pain, highlighting measurement discordance rather than true inefficacy—chronic musculoskeletal sensitization responds better to holistic scales.

It is worth flagging, though only as a speculative interpretive possibility rather than a resolution of the contradiction itself, that trials showing functional improvement without a corresponding change in pain threshold (21) are compatible with—though in no way demonstrate—increased tolerance of discomfort rather than reduced nociceptive input. As emphasized in Section 4.1, this reinterpretation is not supported by any direct measurement in the included studies and should not be substituted for the more parsimonious explanation offered above (measurement discordance between algometry and multidimensional pain scales).

Publication bias risk is moderate, with funnel plots detecting asymmetry in perioperative data adjusted via trim-and-fill, but positive skew in Chinese migraine trials (e.g., 68% female, *p* < 0.001 effects) suggests cultural expectations inflating reports (9, 19). Methodological evolution favors recent meta-analyses (2025) over early pilots (2005–2007), incorporating larger samples and GRADE assessments to mitigate unclear blinding, enhancing reliability over fixed-site comparisons in older sham designs.

As for conflicting findings, we think that the divergence between the null VAS result of Usichenko et al. (10) and the positive pooled perioperative estimates of Zhou et al. (9) is not fully explained by outcome measurement alone; it may also reflect genuine between-protocol variability (single-session vs. retained needling) that a narrative synthesis, unlike a formal meta-regression, cannot statistically adjudicate. Also, on publication bias it should be referred that beyond the funnel-plot asymmetry noted above, the absence of prospective trial registration for several older RCTs [e.g., Usichenko et al. (10, 13)] precludes formal assessment of selective outcome reporting at the study level, and the concentration of positive migraine findings within a small number of Chinese research groups raises the possibility of a “small-study effect” that a larger, more geographically diverse evidence base would be needed to rule out.

Placebo effects and sham-control limitations should also be mentioned, since many sham protocols in the included trials stimulate non-specific auricular sites that may themselves be physiologically active (given the density of somatotopic projections across the ear), which would tend to underestimate rather than inflate true treatment effects, while simultaneously making it difficult to fully exclude non-specific expectation effects as a contributor to the moderate effect sizes observed, particularly for subjective outcomes such as VAS. And we should consider the possible effect of protocol heterogeneity, namely differences in needle type, retention duration, point selection, and number of sessions across the primary RCTs and the meta-analyses that aggregate them (which in some cases include overlapping primary studies, see Section 2.3) limit the comparability of the pooled estimates, and mean that the summary effect sizes in this review should be read as an approximate synthesis of a heterogeneous literature rather than as a single, precise pooled estimate.

A narrative contrast across intervention modality is possible with the data at hand, though it falls short of a formal subgroup analysis. Protocols using retained or indwelling needles over extended periods [e.g., up to 48 h in Usichenko et al. (13); multiple courses in Alimi et al. (17)] tend to report larger and more sustained effect sizes than single-session or brief acupressure protocols [e.g., Wang & Kain (16); 48-hour press needles for anxiety-proxy outcomes], consistent with the dose–response pattern noted in Section 4. Seed/press-needle approaches (15, 17) appear comparably effective to indwelling-needle protocols for chronic and cancer-related pain, though direct head-to-head comparisons within a single trial are absent from the included evidence, and this contrast should therefore be read as descriptive rather than confirmatory.

### 4.2 Practical implications

Given the varying confidence levels established in Section 3.3.6 (Table 4), clinical implications are presented here stratified by certainty of evidence rather than as uniform recommendations across pain types.

Moderate-certainty evidence—auriculotherapy can be reasonably recommended as an adjunct. Perioperative and orthopedic surgical pain represents the setting with the most consistent supporting evidence in this review, with meta-analyzed reductions in analgesic requirement (SMD −0.88, 95% CI −1.29 to −0.46) and VAS at 24 h (MD −0.44, 95% CI −0.72 to −0.17) [Zhou et al. (9)], corroborated by multiple independent primary RCTs (10–13). For adults aged approximately 18–70 undergoing orthopedic or abdominal surgery, preoperative auriculotherapy can be reasonably considered for adoption as a low-risk adjunct to reduce postoperative analgesic requirements, provided it is delivered by trained personnel and positioned explicitly as a complement to, not a substitute for, standard perioperative analgesic protocols.

Limited-to-moderate certainty evidence—auriculotherapy may be considered as an auxiliary measure, with explicit caveats. For chronic back pain and migraine, the evidence base, while directionally consistent (SMD −0.65 to −0.85 for back pain; VAS MD −0.65, 95% CI −0.86 to −0.43 and attack-frequency MD −0.49, 95% CI −0.59 to −0.40 for migraine), is limited by protocol heterogeneity, geographically concentrated samples [migraine evidence is drawn entirely from Chinese trial populations; Zheng et al. (19)], and—for back pain—inconsistency between subjective (BPI, VAS) and objective (algometry) outcome measures within the same trial (21). Clinicians considering auriculotherapy for these conditions should present it to patients explicitly as an adjunct to, rather than a replacement for, established pharmacological and physical-therapy-based management, and should set expectations accordingly given the moderate-to-limited certainty of the underlying evidence.

Limited-certainty evidence—auriculotherapy should be considered exploratory. For cancer-related pain, the evidence is sparser and draws on fewer controlled trials with greater observational elements (17, 20), despite the notable 36% VAS reduction reported at 2 months in the sole dedicated RCT (17). Given this limited certainty, auriculotherapy may be considered as an exploratory adjunct for patients with refractory cancer-related pain who are already stabilized on standard analgesic regimens—particularly in non-metastatic disease, where effects appeared more pronounced (20)—but should not be positioned as a recommended component of standard oncologic pain management pending stronger controlled evidence.

Across all certainty levels, safety findings remain strong and consistent (adverse events confined to mild local reactions in <5%–8% of cases, with no serious events reported), which supports auriculotherapy's viability as a low-risk adjunct wherever it is considered, even where efficacy evidence remains limited. Regulatory and guideline bodies should reflect this evidentiary gradient directly: relatively firm endorsement is defensible for perioperative orthopedic use, while chronic back pain, migraine, and cancer-pain applications should be described in guidelines as adjunctive and evidence-emerging rather than established, pending the large, diverse, standardized trials called for in Section 4.4. As previously noted, these implications derive primarily from adult surgical and oncology populations and should not be extrapolated to pediatric or elderly patients, nor to the anxiety-proxy outcomes reported by Wang and Kain (16), where physiological overlap with pain modulation exists but direct pain outcome data are absent.

### 4.3 Strengths and limitations

Strengths of this review include a comprehensive search across databases, with a good coverage of RCTs and syntheses, and thematic integration that reveals patterns like protocol-dependent effects invisible in isolated studies. The focus on extracted data prioritizes structured outcomes, enhancing precision in numerical reporting. Limitations of included studies encompass moderate RoB from unclear allocation (40%–50% trials) and a moderate to high attrition bias. Many drew from Asian or military populations, reducing generalizability to diverse demographics. No formal meta-analysis was performed due to source heterogeneity.

A further limitation concerns the English-language restriction applied to this review. Because searches were confined to English-language records across PubMed, b-On, Web of Science, Scopus, and SciELO, a substantial body of non-English clinical literature on auriculotherapy—most notably Chinese-language trials, which represent a disproportionately large share of global auriculotherapy research—was systematically excluded. This introduces language-driven selection bias whose direction cannot be determined from the present data: it is possible that excluded non-English trials would reinforce the positive effect directions observed here, but it is equally possible that language-based publication and translation practices favor reporting of positive findings in the English-language literature specifically, which would inflate the apparent effect sizes synthesized in this review. Notably, several of the strongest effect estimates included here [e.g., the migraine findings from Zheng et al. (9)] already derive from research conducted and published by groups based in China but reported in English, raising the related concern that even within the English-language literature, geographic and cultural concentration of researchers may itself constitute a source of bias distinct from, but compounding, the language restriction. Because this review's search and eligibility criteria did not permit a targeted appraisal of the omitted non-English literature, the magnitude and direction of language bias cannot be quantified, and the pooled effect directions reported here should be interpreted as representative of the English-language evidence base specifically, rather than of the global auriculotherapy literature as a whole.

A further, largely unaddressed threat to the external validity of this review concerns the population genetics and the socio-educational and cultural acceptance of acupuncture-based interventions across the settings from which the included evidence derives. The scope of this review was originally framed around deliberately including non-Asian populations to mitigate biases related to population genetics and cultural aspects of treatment acceptance (see Scope Statement); however, the final included evidence base remains predominantly composed of Asian—and specifically Chinese—trial populations [e.g., the entirety of the migraine evidence in Zheng et al. (19)], and a substantial proportion of the perioperative evidence in Zhou et al. (9), alongside a smaller subset of European and North American military/perioperative cohorts (10–13, 16). This concentration raises two distinct, compounding concerns for generalizability.

First, at the biological level, potential population-genetic variability in pain perception, opioid and analgesic metabolism, and autonomic nervous system reactivity—the very physiological pathway proposed to mediate auriculotherapy's effects (7)—has not been systematically characterized or compared across the ethnic and genetic backgrounds represented in the included trials. Should such variability exist, effect sizes derived predominantly from genetically homogeneous samples may not transfer proportionally to populations with different genetic profiles, and this review is not positioned to detect or adjust for such an effect given the absence of genetic or pharmacogenomic reporting in any included study.

Second, and arguably more consequential given the subjective, self-reported nature of most outcome measures used across the included trials (VAS, NRS, BPI), the socio-educational and cultural acceptance of acupuncture and related traditional-medicine modalities differs substantially across the populations and healthcare systems represented. In settings where acupuncture is culturally normalized and clinically integrated (as in much of the Chinese evidence base), patients' prior familiarity with and positive expectancy toward the intervention may amplify placebo-mediated and expectancy-driven components of the observed treatment effect—a mechanism distinct from, but difficult to disentangle from, the autonomic and neuroendocrine pathways proposed as the primary mechanism of action. Conversely, in populations where acupuncture is less culturally familiar, unfamiliar, or viewed with skepticism, comparable expectancy effects—and therefore comparable observed effect sizes—may not materialize even if the underlying physiological mechanism is genuinely present. Sham-controlled designs mitigate but do not eliminate this concern, since patient-level expectancy and cultural framing of the intervention can influence both active and sham arms asymmetrically depending on the surrounding cultural context in which the trial is conducted.

Taken together, these two dimensions—population-genetic variability and differential socio-cultural and educational acceptance of acupuncture—constitute a substantive, currently unquantifiable threat to the external validity of the pooled and narratively synthesized effect estimates reported in this review. The original intention to prioritize non-Asian populations in the search strategy (Scope Statement) was only partially achieved in the final included sample, and future reviews and primary trials should both report participant ethnicity and prior exposure to or familiarity with acupuncture-based interventions as standard covariates, to allow these compounding sources of bias to be empirically examined rather than merely acknowledged.

Relatedly, this review did not conduct formal subgroup analyses. Several dimensions along which auriculotherapy protocols and populations varied across the included studies would merit dedicated subgroup investigation in future systematic reviews or individual-patient-data meta-analyses, including: intervention modality (seed-press vs. indwelling needle vs. laser stimulation), stimulation/needle-retention duration, participant ethnicity and geographic origin, and pain chronicity (acute vs. subacute vs. chronic presentations). The present review's narrative structure allows only qualitative, non-statistical comparison along these dimensions (see Section 3.3 and Section 4.1) and cannot substitute for a properly powered quantitative subgroup analysis.

Two further limitations follow directly from the heterogeneous evidence base underlying this review. First, several primary RCTs synthesized here also contribute data to one or more of the included meta-analyses [e.g. (10, 11) relative to (9)], so the thematic effect estimates in Table 2 should not be read as independent, non-overlapping sources of evidence. Second, sample sizes for individual primary trials ranged as low as *n* = 20 (13), limiting the precision of study-level estimates even where pooled meta-analytic estimates from larger aggregated samples appear more precise. Taken together with the mix of study designs (primary RCTs alongside existing systematic reviews, meta-analyses, and a scoping review) and populations, these factors mean the review's synthesis should be regarded as a structured narrative overview rather than a quantitatively pooled meta-analytic effect.

### 4.4 Gaps and future directions

Key gaps include sparse data on long-term efficacy beyond 2 months, with most trials (e.g., perioperative meta-analyses) assessing only 24–48 h (9), leaving sustained chronic pain relief unaddressed despite promising 15-day follow-ups in back pain (14). Mechanistic evidence is superficial, mentioning autonomic/neuroendocrine pathways without biomarkers like endorphin levels or fMRI validation (7), hindering causal attribution.

The discussion above (Section 4.1) offers a post-hoc, hypothesis-generating interpretation linking select behavioral outcomes to the construct of pain acceptance; this interpretation is not supported by direct measurement in any included study and must not be mistaken for an established finding. Future trials should formally test this hypothesis by prospectively incorporating validated pain-acceptance instruments (e.g., the CPAQ) as defined primary or secondary outcomes, rather than inferring acceptance-related change retrospectively from proxy measures such as analgesic consumption or interference scores. Only such prospective, instrument-based verification would allow readers to distinguish a genuine effect of auriculotherapy on pain acceptance from the tentative, indirect inference offered in this review.

Beyond pain acceptance specifically, future systematic reviews and individual-patient-data meta-analyses would benefit from formally testing the subgroup dimensions outlined in Section 4.3—auriculotherapy modality (seed-press, indwelling needle, laser stimulation), stimulation duration, participant ethnicity, and pain chronicity—as pre-registered moderators, which the present narrative synthesis could only address descriptively (Section 4.1).

Underrepresentation of non-Asian, pediatric, or elderly populations persists, as migraine syntheses are China-centric (68% female adults) (19), and no studies target neuropathic pain directly. Contradictions in sham equivalence [e.g., no VAS superiority in knee RCT (10)] remain unresolved due to inconsistent controls. To answer the research question for broad pain populations, large RCTs with diverse demographics (e.g., >500 participants across ages/genders) are needed, using standardized protocols (e.g., fixed Shenmen stimulation) and harmonized VAS/NRS outcomes over 6–12 months.

Methodological improvements should incorporate objective measures like algometry alongside subjective scales, advanced blinding (e.g., laser sham), and subgroup analyses by pain chronicity. Targeted research on underrepresented contexts, such as community-based chronic pain in low-income groups, would enhance applicability.

Future research should prioritize double-blind randomized controlled trials (RCTs), in which both participants and outcome assessors remain unaware of treatment allocation. Blinding remains a persistent methodological challenge in acupuncture research, since practitioners cannot be blinded to needle placement, and several of the risk-of-bias concerns identified in this review (Section 3.2) stem from unclear or absent blinding of participants and outcome assessors. Adopting validated sham-blinding techniques (e.g., non-penetrating or blunt-tip sham needles, laser sham stimulation) alongside independent, blinded outcome assessment would reduce the risk of performance and detection bias and strengthen confidence in the observed effect sizes, particularly for subjective, self-reported outcomes such as VAS and NRS.

## 5 Conclusion

Auriculotherapy exhibits moderate efficacy in reducing pain across acute postoperative, chronic musculoskeletal, migraine, and cancer contexts, with meta-analytic support for VAS decreases of −0.44 (95% CI: −0.72 to −0.17) perioperatively and −0.65 (95% CI: −0.86 to −0.43) in migraines among adults, alongside SMD −0.65 to −0.85 for back pain intensity and an analgesic-requirement SMD of −0.88 (95% CI −1.29 to −0.46) in the perioperative setting. These benefits, sustained in subsets like 36% VAS reductions at 2 months for cancer patients (*p* < 0.0001), stem from consistent trial designs favoring needle-based protocols over single sessions, though evidence draws primarily from surgical and Asian cohorts partially matching broader pain populations, necessitating cautious extrapolation to non-clinical or diverse groups. This synthesis combines primary RCTs with existing systematic reviews, meta-analyses, and a scoping review through narrative rather than quantitative pooling; readers should therefore weigh the summary effect sizes above as an indicative overview of a methodologically and demographically diverse evidence base, rather than as a single precise pooled estimate, when considering the strength of the conclusions that follow.

Safety is unequivocally strong, with adverse events confined to mild local issues in <5%–8% of cases, positioning it as a viable non-pharmacological adjunct. Yet, the most critical uncertainty lies in long-term outcomes and mechanistic depth—current data hint at autonomic modulation but lack confirmatory biomarkers, leaving questions about durability in entrenched chronic pain unresolved.

Addressing this through rigorous, population-diverse trials could solidify auriculotherapy's role, potentially transforming pain management by curbing opioid dependency and improving quality of life for millions, with profound implications for integrative care in an era of escalating chronic disease burdens.

## Data Availability

The datasets presented in this study can be found in online repositories. The names of the repository/repositories and accession number(s) can be found below: N/A.
